# Ethical Tensions in FAA Mental Health Policies for Aspiring Hobbyist Pilots

**DOI:** 10.1192/j.eurpsy.2026.11643

**Published:** 2026-07-31

**Authors:** M. Hurta, E. M. Jetter, T. Waldmann, B. Carr

**Affiliations:** 1 College of Medicine; 2 Department of Psychiatry, University of Florida, Gainesville, United States

## Abstract

**Introduction:**

In the United States (U.S.), mental health management for aspiring hobbyist pilots presents a complex ethical landscape where individual well-being intersects with public safety and regulatory compliance. The U.S.Federal Aviation Administration’s (FAA) policies on psychiatric conditions impose strict reporting requirements that can create conflicts between personal treatment decisions and regulatory oversight.

**Objectives:**

This work explores the ethical tensions embedded in U.S. FAA mental health policies. Through the lens of autonomy, utilitarianism, and deontological ethics, we analyze how these principles shape the regulatory framework, highlight the consequences for aspiring pilots, and propose ethically balanced policy recommendations.

**Methods:**

We conducted a critical ethical analysis of U.S. FAA policies through the lens of three foundational ethical theories. Illustrative real-world cases were used to demonstrate practical dilemmas faced by aspiring pilots with depressive symptoms who sought treatment while attempting to comply with or circumvent FAA regulations.

**Results:**

Both cases revealed ethical conflicts between effective treatment and regulatory compliance. One aspiring pilot, a middle-aged physician with dysthymia, declined antidepressants despite his psychiatrist’s recommendation and pursued transcranial magnetic stimulation (TMS) to avoid disclosure; his mood remained low and suicidal thoughts developed. He eventually accepted SSRI therapy, disclosed his treatment and obtained certification. Another professional with newonset major depression similarly selected TMS to bypass reporting requirements; his depressive symptoms persisted until he began cognitive behavioral therapy and disclosed his condition. These cases demonstrate how rigid policies incentivize treatment invisibility over clinical efficacy, creating perverse incentives that may compromise both health outcomes and safety.

**Conclusions:**

U.S. FAA mental health policies highlight the tension between safeguarding public safety and respecting individual mental health needs. The cases underscore the ethical complexity of treatment choices when disclosure risks personal goals. We propose adaptive mental health criteria, collaborative oversight and confidential support systems to reduce perverse incentives. A more nuanced regulatory framework is needed to balance public safety with ethically robust, compassionate care.

**Disclosure of Interest:**

None Declared

